# Estrogen induced downregulation of gene expression and cell biological processes critical for genital tubercle formation via DNA methylation

**DOI:** 10.1186/1756-8935-6-S1-P33

**Published:** 2013-03-18

**Authors:** Jia-Xin Jiang, Karen J Aitken, Tyler P Kirwan, Sevan Hopyan, Darius J Bägli

**Affiliations:** 1 Department of Physiology, University of Toronto, Toronto, ONo, Canada, M5S 1A8; 2 Department of Urology, Hospital for Sick Children, Toronto, ON, Canada, M5G 1X8; 3 Research Institute, Department of Developmental & Stem Cell Biology Hospital for Sick Children, Toronto, ON, Canada, M5G 1X8; 4 Division of Orthopaedics, Department of Surgery, Hospital for Sick Children, Toronto, ON, Canada, M5G 1X8

## Background

Hypospadias is one of the most common genital birth defect, characterized by improper closing of the urethral folds during development and resulting ectopic opening of the urethra. Xenoestrogen (XE) exposure increases the rate of hypospadias in both animal models and in humans, in association with gene down regulation.[1,2] While many genes (e.g. SHH, WNT5A, CTNNB1, HOXA13) are critical for genital tubercle (GT) and urethral development in genetically deficient models, the role of such genes in response to estrogen exposure in GTs has been less studied. While XE exert known epigenetic effects, the mechanism involved is not clear. This study examines the epigenetic effects of XE on GT developmental genes, as well as on DNA methylation enzymes (DNMTs) involved in epigenetic downregulation.

## Material and methods

The BJ normal human foreskin fibroblast cell line was used to model GT mesenchyme. They were stimulated every 24 hours with 100 nM of diethylstibestrol (DES) for 6, 24, 48 and 120 hours +/- 2-dexoxy-5-azacytidine (aza), a DNMT inhibitor. Real-time PCR was performed to examine expression of candidate genes (WNT5A, HoxA13, HoxA10, DNMT-1, -3Aand-3B, and others), normalized to rpl19 or gapdh.

## Results

DNMTs were all temporally upregulated significantly by DES treatment: 75% at 6 hours for DNMT3A (p<0.05), >2 fold at 120 hours for DNMT1 (p<0.005), and >5 fold at 6, 24 and 120 hours for DNMT3B. Hox and WNT5A gene expression was conversely downregulated by DES and partially restored by aza. HoxA13 was downregulated between 70-92% at 24, 48 and 120 hours of DES treatment (p<0.05). Aza recovered expression of HoxA13 by 3-fold (p<0.04) by day 5. At 48 hours, Wnt5A also showed downregulated expression, which was increased 5.6-fold by aza (p<0.02), though at other time points was not similarly affected by DES.

## Conclusions

DES treatment activates the epigenetic machinery, via increased DNMT expression. This epigenetic response is accompanied by a down regulation of WNT5A and HoxA13, genes critical for GT formation. This downregulation is dependent on DNA methylation as DNMT inhibition with aza partially restores HoxA13 and WNT5A expression. These results suggest that environmental XE may act epigenetically to induce long-term alterations in genes crucial for genital development.
